# Morphological profiling data resource enables prediction of chemical compound properties

**DOI:** 10.1016/j.isci.2025.112445

**Published:** 2025-04-16

**Authors:** Christopher Wolff, Martin Neuenschwander, Carsten Jörn Beese, Divya Sitani, Maria C. Ramos, Alzbeta Srovnalova, María José Varela, Pavel Polishchuk, Katholiki E. Skopelitou, Ctibor Škuta, Bahne Stechmann, José Brea, Mads Hartvig Clausen, Petr Dzubak, Rosario Fernández-Godino, Olga Genilloud, Marian Hajduch, María Isabel Loza, Martin Lehmann, Jens Peter von Kries, Han Sun, Christopher Schmied

**Affiliations:** 1 Leibniz-Forschungsinstitut für Molekulare Pharmakologie im Forschungsverbund Berlin e.V. (FMP), Campus Berlin-Buch, Robert-Roessle-Str. 10, 13125 Berlin, Germany; 2 Fundación MEDINA, Centro de Excelencia en Investigación de Medicamentos Innovadores en Andalucía. Parque Tecnológico Ciencias de la Salud, Avda. del Conocimiento 34, 18016 Armilla, Granada, Spain; 3 Institute of Molecular and Translational Medicine, Palacky University and University Hospital in Olomouc, Faculty of Medicine and Dentistry, Hněvotínská 5, 77515 Olomouc, Czech Republic; 4 Biofarma Research Group, Centro de Investigación CIMUS, Departamento de Farmacología, Facultad de Farmacia, Instituto de Investigaciones Sanitarias IDIS, Universidade de Santiago de Compostela, 15706 Santiago de Compostela, Spain; 5 EU-OPENSCREEN ERIC, Campus Berlin-Buch, Robert-Roessle-Str. 10, 13125 Berlin, Germany; 6 CZ-OPENSCREEN: National Infrastructure for Chemical Biology, Institute of Molecular Genetics of the Czech Academy of Sciences, Vídeňská 1083, 14220 Prague 4, Czech Republic

**Keywords:** Chemistry

## Abstract

Morphological profiling with the Cell Painting assay has emerged as a promising method in drug discovery research. The assay captures morphological changes across various cellular compartments enabling the rapid prediction of compound bioactivity. We present a comprehensive morphological profiling resource using the carefully curated and well-annotated EU-OPENSCREEN Bioactive compounds. The data were generated across four imaging sites with high-throughput confocal microscopes using the Hep G2 as well as the U2 OS cell lines. We employed an extensive assay optimization process to achieve high data quality across the different sites. An analysis of the extracted profiles validates the robustness of the generated data. We used this resource to compare the morphological features of the different cell lines. By correlating the profiles with overall activity, cellular toxicity, several specific mechanisms of action (MOAs), and protein targets, we demonstrate the dataset’s potential for facilitating more extensive exploration of MOAs.

## Highlights

• Cell Painting dataset based on 2464 EU-OPENSCREEN Bioactive compounds
• Hep G2 datasets generated from four different imaging sites
• Extensive assay optimization within and across sites yields high quality and reproducibility
• Exploration of compound bioactivity and prediction of mechanisms of action

## Graphical abstract

**Figure:**
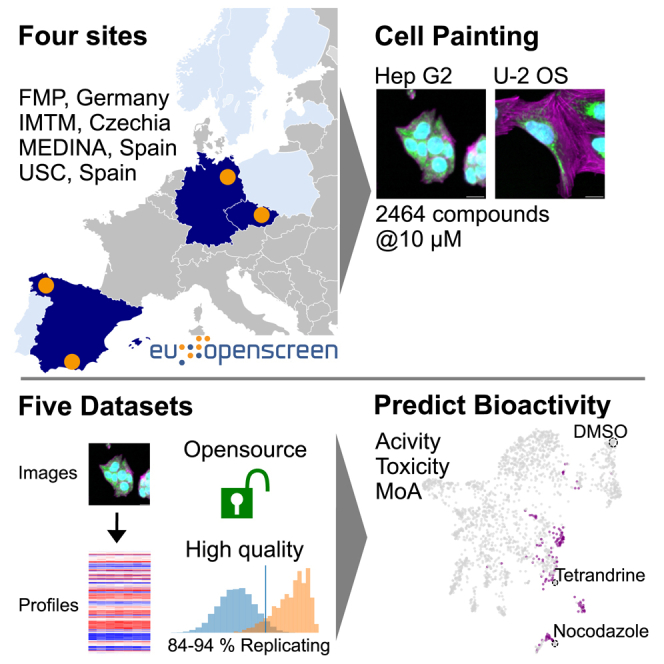

## Introduction

High-throughput morphological profiling of small molecule libraries, using the Cell Painting assay, has received increasing attention in drug discovery research.^1^^,^^2^^,^^3^ Compared to conventional high-throughput screening of a single biological target, morphological profiling with high content imaging offers the advantage of identifying multiple biological activities of small chemical compounds simultaneously, promising to substantially accelerate the early stage of the drug discovery process. Moreover, this method enables to predict toxicity and more specifically the mechanism of action (MOA) of drug-like compounds at the cellular or subcellular levels in a non-invasive manner.^4^ A typical Cell Painting assay uses six fluorescent stains imaged over multiple channels, revealing morphological changes upon perturbation of cells in eight major cellular compartments, namely DNA, cytoplasmic RNA, nucleoli, actin, Golgi apparatus, plasma membrane, endoplasmic reticulum, and mitochondria.^1^

To analyze the morphological changes in cells, induced by small chemical compounds in these different cellular compartments, high dimensional image features need to be extracted from the generated images.^5^^,^^6^^,^^7^ In classical Cell Painting analysis, hundreds of handcrafted image features are extracted using computational tools such as CellProfiler.^8^ The extracted features serve as fingerprints or profiles that quantitatively characterize the induced cellular phenotypes. Dimension reduction and clustering of the profiles enable the identification of biological activity of uncharacterized chemical compounds. These methods have been successfully employed in recent years to identify chemical probes and drug-like molecules for various biological targets^9^^,^^10^^,^^11^^,^^12^ such as the Sigma 1 receptor antagonist^13^ and mitotic kinesin inhibitors,^14^ as well as for treating SARS-CoV-2 infections.^15^

For an exhaustive characterization of small molecules and their activity, it is crucial that large high-quality data sources exist that systematically assay under as many experimental conditions, e.g., compound concentrations and cell models, as possible. One large data source is the Joint Undertaking for Morphological Profiling (JUMP) Cell Painting Consortium, which has very recently published and released a large collection of the Cell Painting data using U-2 OS cells stemming from a joint effort of various academia and industrial partner sites.^16^^,^^17^ Here, we present a Cell Painting dataset of the EU-OPENSCREEN Bioactive compound set. EU-OPENSCREEN is a European Research Infrastructure Consortium (ERIC) dedicated to accelerating the discovery of small molecule compounds for new biological targets by providing academic research groups with open-access to high-throughput screening technologies.^18^ EU-OPENSCREEN hosts multiple compound collections that contain in total more than 100,000 compounds. The largest library is the European Chemical Biology Library (ECBL) composed of commercially available compounds selected to cover a diverse chemical space.^19^ The Bioactive compound set is part of the Pilot Library and consists of 2,464 Bioactive compounds carefully chosen for their diverse biological activity, including 681 approved drugs and 385 highly selective probes. Thus, this set serves as an ideal reference for the Cell Painting assay.

Within this study, we generated multiple Cell Painting datasets of the EU-OPENSCREEN Bioactive compound set over four different imaging sites, primarily using the Hep G2 cell line and for comparison with other datasets in the Cell Painting community in the U-2 OS cell line. Image acquisition was performed using high throughput confocal microscopes. To validate our assay, we performed multiple replicates, which demonstrated the high reproducibility and robustness of the method. To facilitate further research in this field, we have made the data freely available via the Cell Painting Gallery,^20^ which will inspire the development of novel computational approaches for identifying the biological activity of chemical compounds. Moreover, this dataset will serve as an important reference for future high-throughput screening based on the larger EU-OPENSCREEN collection.

## Results

### EU-OPENSCREEN Bioactive compound set

**Figure 1:**
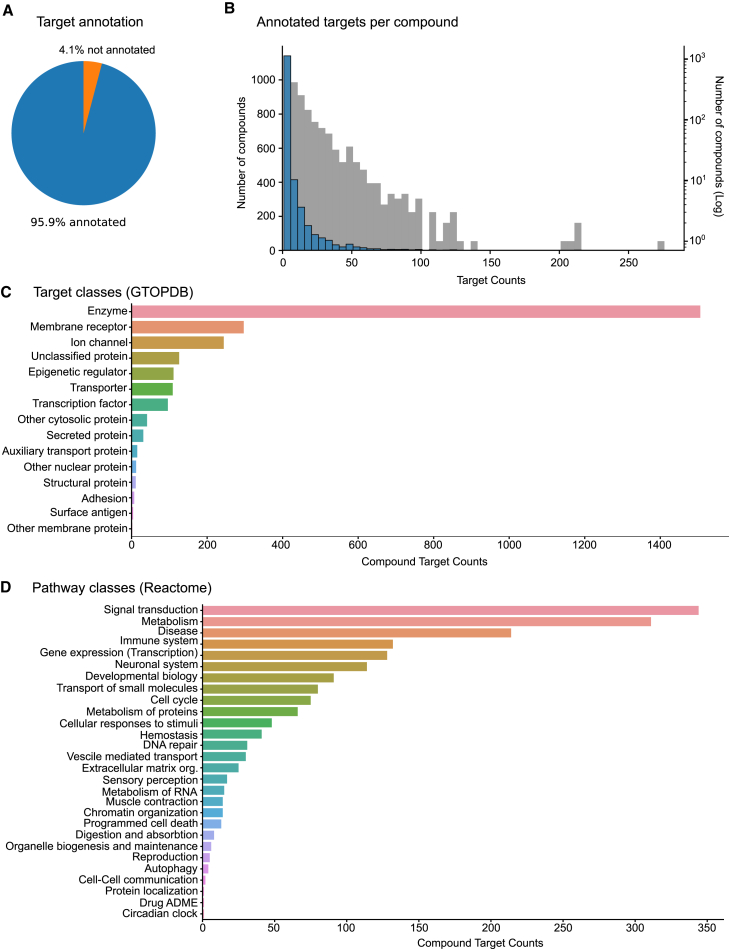
EU-OPENSCREEN Bioactive compound set (A) Almost all of the 2,464 compounds of the bioactive set have at least a single annotated target. (B) Histograms of the number of compounds over the number of targets (bin size of 5, log scale in gray). (C) The total number of different compound targets annotated in the compound set was 2,841. These annotated compound targets range over a diverse number of target classes based on their GTOPDB annotation. (D) The compound targets are also implicated in a diverse range of pathway classes based on their Reactome annotation.

The EU-OPENSCREEN Bioactive compounds are a densely annotated small compound set that consists of 2,464 compounds, with 96% of the compounds having at least a single annotated target (Figure 1A). This set is enriched for approved drugs, chemical probes, and compounds with known MOA (see STAR Methods: bioactive set). In total the set is linked to 2,841 different compound targets. Most compounds have multiple targets annotated in the literature, with a median number of six targets per compound and one compound with a maximum of 272 targets annotated, demonstrating the capability of small compounds to interact with multiple targets (i.e., polypharmacology, Figure 1B). The set was generated with the intention of a wide proteome coverage and therefore the targets range over many different target classes (Figure 1C; Table S1) and are involved in many different pathways (Figure 1D; Table S2). A similarity comparison of the compound structure between the EU-OPENSCREEN Bioactive compounds as well as the JUMP-CP compounds^16^ revealed that 1,210 compounds are in common between these two compound sets (Table S3).

### Cell painting assay

**Figure 2:**
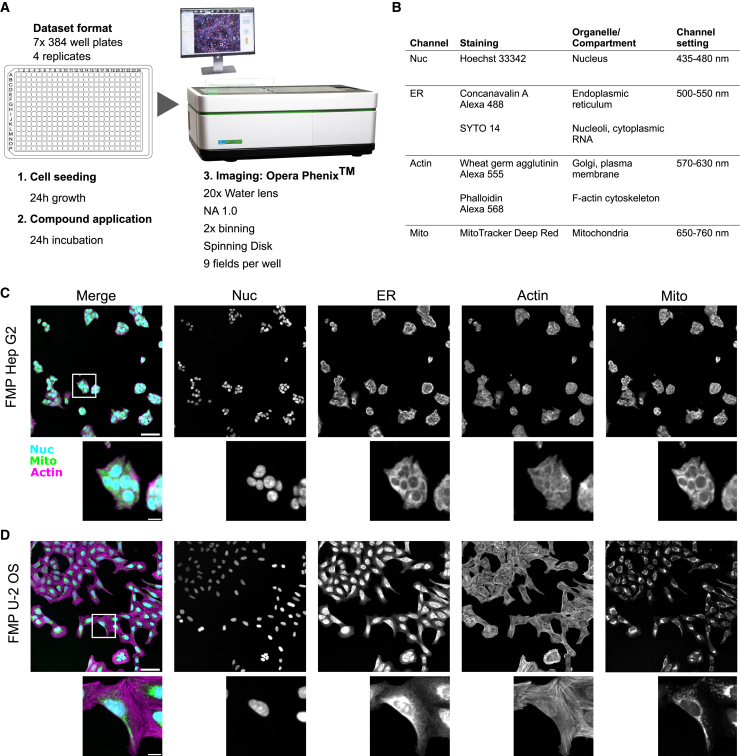
Cell Painting assay performed on EU-OPENSCREEN Bioactive compound set (A) Cell Painting approach using high-throughput confocal imaging. Image of Opera Phenix, credit and © PerkinElmer. (B) Four imaging channels with the associated staining labeling different cellular compartments. (C) Composite image of the nucleus (Nuc, Cyan), mitochondria (Mito, green) and the actin (Actin, magenta) channels. Individual channels in gray-scale of Hep G2 cells treated with DMSO are shown; a cluster of cells is shown below. (D) Merge of the nucleus (Nuc, Cyan), mitochondria (Mito, green), and the actin (Actin, magenta) channels and the individual channels in gray-scale of U2OS cells treated with DMSO. Enlarged single cell cropped close to the center of the field of view is shown below. Note that the individual channels between the different cell lines were adjusted using different brightness contrast settings. (C and D) Scale bars in merge panels correspond to 100 μm in the full field of view and 20 μm in the crop.

To characterize the EU-OPENSCREEN Bioactive compound set we employed a Cell Painting assay based on an established protocol.^1^ The assay was carried out at four different imaging sites (FMP - Leibniz-Forschungsinstitut für Molekulare Pharmakologie, Germany; IMTM - Institute of Molecular and Translational Medicine; MEDINA - Fundación MEDINA; USC – Universidad de Santiago de Compostela, Spain) with the 2,464 compounds of the set distributed on seven 384-well plates. The assay was performed over four replicates per dataset from the different sites. After cell seeding, the cells were grown for 24 h before being incubated for 24 h with the compounds at 10 μM concentration (Figure 2A). DMSO was used as a negative control and reference for the plate normalization. We used tetrandrine and nocodazole at 5 μM concentration as positive controls since these compounds show a strong phenotypic response in different cell lines.

The cells were then fixed and stained with six stains labeling different cellular compartments. Imaging acquisition was performed using spinning disk confocal systems over nine fields per well (Figure 2A). The six cellular stains were then acquired in four separate channels (Figure 2B). The assay was performed primarily on the Hep G2 cell line generating 387,072 images per dataset (Figure 2C). One site (FMP) additionally applied the Cell Painting assay with the bioactive set on the U-2 OS cell line (Figure 2D), a cell line with many already existing Cell Painting datasets.^16^^,^^21^^,^^22^^,^^23^^,^^24^ We were able to directly compare our data from the Hep G2 cell line with data based on this very widespread cell line and will enable the Cell Painting community to compare the Cell Painting data from the Bioactive compound set with their own datasets.

Overall, we aimed to produce high quality data, increasing comparability across sites keeping the experimental conditions as consistent as technically feasible. To achieve this, we performed an extensive evaluation and validation process. First, we selected suitable imaging sites based on submitted proposals and validation data, which were quantitatively evaluated by two external reviewers (see STAR Methods: Assay optimization and standardization, Data S1). Further, we optimized the protocol to reduce variability across sites. In our experience the cell culture as well as staining conditions were responsible for most of the variability and thus, we supplied the same cell culture serum, cells and the same lot fluorescent dyes centrally. For practical and technical reasons some parameters remained different across sites. For instance, the four imaging sites employed three different microscopy systems (see STAR Methods: image acquisition). Since the same compound dataset has been acquired with four replicates, the dataset will provide an opportunity to study and develop methods to overcome experimental variability during downstream processing.

### Extraction of morphological profiles

For analyzing the image data we performed an established JUMP-CP CellProfiler based pipeline^16^ extracting 2,977 handcrafted image features within single cells based on three cell areas (nucleus, cell, cytoplasm). The features based on single cells were filtered using a histogram-based outlier selection (HBOS),^25^ as well as missing and infinite values. Single cell features were aggregated using a median per well. Heatmaps for each plate were plotted as a visual quality control (see QC_Platemaps - Zenodo: https://doi.org/10.5281/zenodo.14776021)*.* For investigating artifacts that may arise from dispensing cells unevenly or uneven cell growth, the cell count per well was plotted (“Metadata_Object_Count”). Typical observed artifacts were dispenser stripes, arising from a badly primed dispenser cassette. Most plates showed no issues with cell seeding/cell growth and only a few plates had an elevated overall cell number.

For artifacts that may arise in the first staining step with live cells and the mitotracker staining, the mean intensity of the cytoplasm segmentation of the Mito channel was used (“Cyto_Intensity_MeanIntensity_Mito”). Typical observed artifacts were rim and edge effects, probably due to lower humidity in the outer wells during the 48-h cell growth incubation step, which may lead to cellular stress conditions. These rim effects in the Mito channel were consistently noticeable in the FMP und USC datasets but were not detected in the MEDINA and IMTM datasets. To reduce such effects thermal variation and differences in evaporations should be kept to a minimum by using for instance spacer plates in plate stacks filled with water, additional reservoirs in the incubator as well as water vapor permeable membranes for sealing plates.

For quality control of the second staining step with fixed cells using the staining mixture that combines the dyes for the ER, Actin, and Nuc channel, following plots were created: the mean intensities of the cytoplasm segments for Actin and ER channel (“Cyto_Intensity_MeanIntensity_AGP” and “Cyto_intensity_MeanIntensity_ER”). Additionally, based on the segmentation of the nuclei, the mean intensities of the Nuc and ER channel was plotted (“Nuc_Intensity_MeanIntensity_DNA” and “Nuc_Intensity_MeanIntensity_ER”). Typical artifacts observed in the second staining step are slight left-to right or top-down plate drifts (i.e., lower or higher intensity in a channel along the rows or columns of a plate), likely arising from the reading direction of the microscope. These heatmaps in general indicate no plate-based artifacts, with rare cases of drift in the AGP channel. Additionally, calculating the median of the four replicates for most channels effectively reduces the majority of these artifacts.

**Figure 3:**
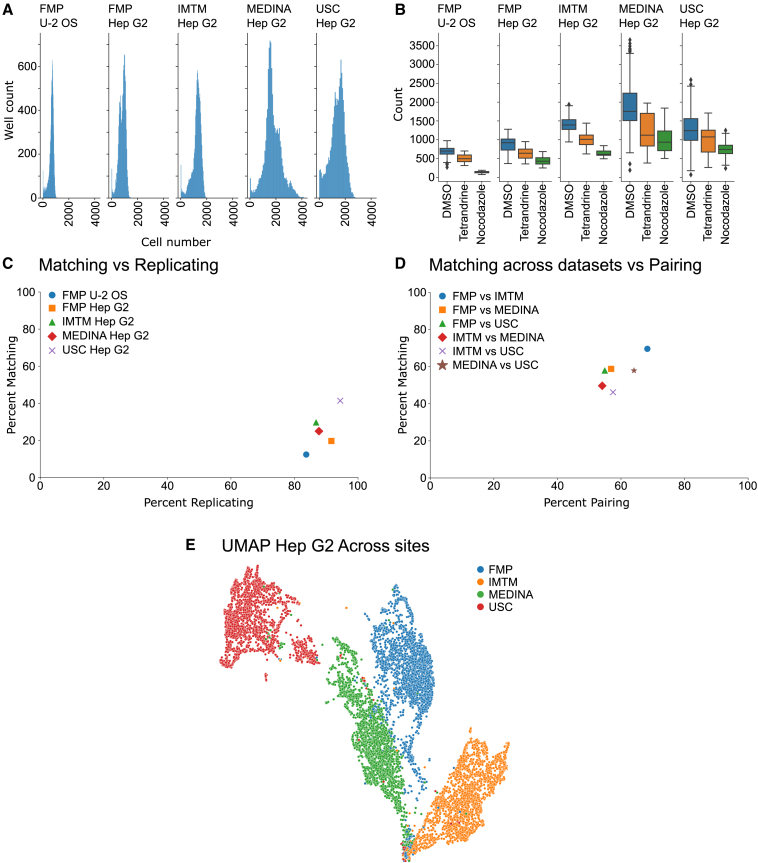
Quantitative quality assessment of dataset and comparison across cell lines (A) Raw cell counts over all plates and wells in U-2 OS and Hep G2 from four different sites. (B) Cell counts in negative and positive controls in U-2 OS and Hep G2 cells from four imaging sites. (C) Percent replicating plotted against percent matching of all presented datasets. (D) Percent matching across datasets versus percent pairing of all Hep G2 datasets. (E) 2D UMAP for assessing batch effects of the datasets from the FMP, IMTM, MEDI and USC. Datasets were combined after normalization. Feature reduction was performed on combined dataset. (B) Data are represented as boxplots with the middle line of the plot corresponding to the median. The lower end of the box to the 1^st^ quartile and the upper end to the 3^rd^ quartile range of the data i.e., the interquartile range. The upper and lower whiskers correspond to the upper and lower 1.5 times the interquartile range with the dots corresponding to the outliers beyond this range.

Analyzing the raw cell numbers per well revealed that most of the wells show an increase of cell number from the initial number of seeded cells. A small number of wells were exhibiting a reduction in cell number, indicating severe cytotoxic effects of some compounds in the set at the given concentration (Figure 3A). The median cell number, acquired from the 9 fields per well and after cell filtering, was varying between the different imaging sites from 691 to 1,659 cells (Figure 3A; Table 1). The pairwise correlation of the cell number per compound across the different imaging sites confirms that the effect of the compounds on the cell number follows a similar trend across the sites i.e., compounds high toxicity show high toxicity in both compared sites (Figure S1).

**Table 1:** Number of compounds per processing step in each dataset

Site	Cell line	Median Cell count	MAD cell count	All cpds	Toxic	% toxic	low active	% low active	Non replicating	% non-replicating	After filtering
FMP	U-2 OS	691	91	2,467	177	7%	690	28%	261	11%	1,339
FMP	Hep G2	863	180	2,467	103	4%	1,503	61%	72	3%	789
IMTM	Hep G2	1,293	178	2,467	118	5%	1,673	68%	89	4%	587
MEDINA	Hep G2	1,659	347	2,467	80	3%	1,542	63%	104	4%	741
USC	Hep G2	1,455	347	2,467	92	4%	1,768	72%	34	1%	573

We also visualized the cell numbers of the control wells, showing the expected reduction of average cell numbers in the positive controls compared to the negative controls, with nocodazole showing a larger overall reduction in cell number than tetrandrine in both cell lines (Figure3B). Of note is the more pronounced reduction in the overall cell number at the given concentration in the U-2 OS dataset (Figure 3B: FMP U-2 OS), which was much more pronounced as compared to the Hep G2 cells from all imaging sites (Figure 3B: Hep G2). The potent cytotoxic effect of nocodazole in U-2 OS cells was also evident through the increased number of dead cells, very small and round cells with small and bright nucleus, in U-2 OS cells (Table 2; Figure S2B) as compared to Hep G2 cells (Table 2; Figure S3B).

**Table 2:** Analysis of dead cells in controls of the FMP U-2 OS and Hep G2 data

Cell line	Treatment	# dead cells	# total cells	% dead cells
U-2 OS	DMSO	969	444,867	0.2
U-2 OS	Nocodazole	1,516	8,206	18.5
U-2 OS	Tetrandrine	86	16,699	0.5
Hep G2	DMSO	1,003	922,096	0.1
Hep G2	Nocodazole	517	48,215	1.1
Hep G2	Tetrandrine	309	32,198	1.0

For further analysis we reduced the total 2,977 features to around 700 features per dataset (Table S4) based on established feature reduction approaches.^1^^,^^5^^,^^16^^,^^26^ Feature reduction was performed by removing features with missing values, low variance, outlier features and most importantly reducing highly correlated features. We observed that the adopted feature selection, generated a feature set that was balanced across various feature types and cellular areas (see Supplementary*_*Material - Zenodo*:* https://doi.org/10.5281/zenodo.14776021). For a qualitative assessment, we visualized the median consensus morphological profiles over the replicates per plate for the positive controls. As the positive controls are present across the seven plates of the Cell Painting assay, the visualization showed that the selected features were highly consistent across the different plates and produced distinct patterns for the two different control compounds (Figures S4A and S4B). Consistency across replicates in each plate was further confirmed quantitatively by comparing the correlation of the controls over replicates over each plate (see QC*_*Correlation_Controls - Zenodo*:* https://doi.org/10.5281/zenodo.14776021).

### Toxicity, activity, and reproducibility of imaged compounds

We then proceeded with a general characterization of the datasets and the compound set by assessing the overall activity, cytotoxicity, as well as the reproducibility of the compound profiles. Highly toxic compounds, albeit bioactive, usually show nonspecific MOAs.^27^ Conversely, compounds with very low activity, at the given concentration in the specific cell line, may lead to unspecific morphological profiles. Toxicity was assessed based on cell number, where compounds were defined as acting toxic if they reduced the cell number to below 2.5 standard deviations of the median cell number of the entire dataset. Here, we show that only 3–7% of compounds in the bioactive set had a toxic effect on the cells, with Hep G2 showing a slightly higher resistance against the toxic effects of compounds at the used concentration (Table 1).

For assessing the activity of compounds, we used the induction score,^23^ defining compounds exhibiting lower activity in the given cell line at the used concentration when less than 5% of their features were positively or negatively deviating compared to the DMSO negative control. Using this induction score 28% of the total number of compounds in the case of the U-2 OS dataset show lower activity. In the case of the Hep G2 datasets the z-scores were in general much lower compared to the U-2 OS cells. This difference is also apparent when applying the same induction threshold, which describes 61–72% of compounds in the Hep G2 cell lines as having lower activity (Table 1). The fact that the Hep G2 cell line shows a much lower response than other cell lines has been recently described.^28^ However, one must note that the induction threshold applied here to assess activity was optimized for U-2 OS cells using a handpicked feature set.^23^ In this work, we aimed to specifically compare the Hep G2 datasets to the U-2 OS dataset and thus did not change this threshold. Other analysis approaches might require adjusting such an analysis to the specific cell lines and feature set and used concentration.

To assess the quality of the datasets quantitatively we computed the percentage of replicating as well as matching compounds within the datasets after applying toxicity and induction filters. This analysis revealed that the morphological profiles are highly replicating in both cell lines over the different imaging sites with datasets showing a percent replicating from 84 to 94% (Figures 3C and S5A–S5E). Thus, only 1–11% of compounds exhibit non-replicating profiles from the original 2467 compounds before toxicity and induction filter (Table 1). The percent matching scores ranged from 12 to 41% (Figures 3C and S5F–S5J). The percent replicating as well as percent matching scores of these datasets are in line with the published scores of comparable datasets.^17^

For further quality control of the dataset, we also filtered the non-replicating compounds and performed dimensionality reduction using Uniform Manifold Approximation and Projection (UMAP).^29^ This allows the visual detection of any batch effects within the datasets from each imaging site.^30^ Indeed, the U-2 OS data produced at the FMP imaging site exhibits some batch effects (Figures S6A and S6B). The Hep G2 datasets from each of the four imaging sites show no apparent batch effects using this visualization (Figures S6C, S6D, and S7A–S7F).

A key challenge for collecting large datasets across different laboratories is combining datasets due to batch effects across imaging sites.^30^ These are the results of slight variations in the application of the protocol and differences in technical equipment such as different microscopes. To assess the impact of batch effects across sites we directly compared the Hep G2 datasets across the different imaging sites (Figure 3D). We performed a modified percent replicating score that we defined percent pairing as the comparison of the same compounds only involved a pairwise comparison across the datasets using the consensus profiles and to differentiate it from technical replication and other metrics such as percent matching.^31^ We also applied percent matching across modalities metric.^17^ This revealed percent pairing scores ranging from 54 to 68% (Figures S8A–S8F) as well as percent matching across datasets ranging from 46 to 70% (Figures 3D and S8G–S8L). Although more than half of compounds yield highly similar profiles, a direct combination of such data cannot be achieved without further processing (Figure S3E). Further standardization of such data will necessitate an increase in automation as well as increased standardization of imaging equipment and the image acquisition.^32^^,^^33^ Additionally, the development and application of batch correction methods will also be vital for working with large datasets generated by multiple laboratories.^30^

### Comparison of Hep G2 and U-2 OS

**Figure 4:**
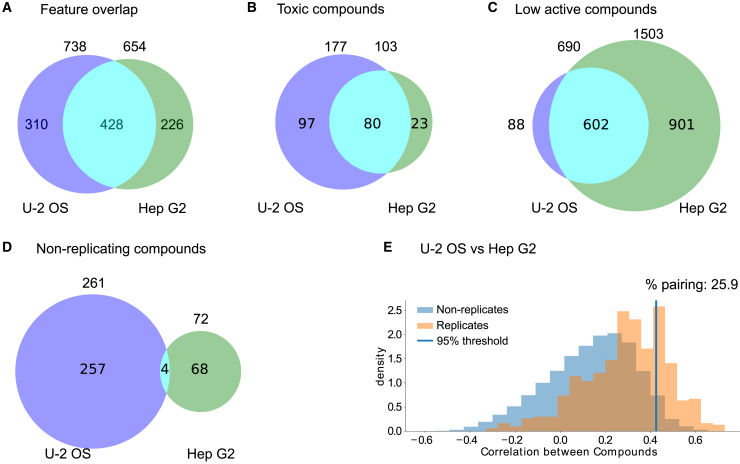
Comparison across cell lines based on FMP datasets (A) Number of image features after feature reduction and feature overlap between U-2 OS and Hep G2 cells. (B) The number of toxic compounds and overlap between different cell types. (C) Number of compounds that did not pass the induction filter (lower active compounds) and the overlap between U-2 OS and Hep G2 cells. (D) Number of non-replicating compounds and overlap between different cell lines. (E) Percent pairing of 564 non-toxic and highly active compounds across U-2 OS and Hep G2 datasets from the FMP imaging site compared over 427 overlapping features.

We further analyzed the available datasets by comparing the U-2 OS and the Hep G2 cell lines. For a direct comparison, we focus on biologically relevant differences between these cell lines. We thus compare the data from one imaging site to exclude confounding factors that arise from any technical differences (e.g., microscopy, precise staining protocols and devices). Directly comparing the features after the same feature extraction pipeline was applied to the data using independent feature reduction revealed that most features are consistent across the different cell lines (Figure 4A). The morphological profiles of the 428 overlapping features in the positive controls revealed that some features exhibit consistent responses across cell types, while many others displayed varying responses (Figures S4C and S4D), in line with previous findings in the literature.^34^

We have already noted that Hep G2 cells exhibited greater resistance to toxic compounds at the given concentration when compared to U-2 OS cells (Figures 3A and 3B; Tables 1 and 2). We subsequently investigated the extent of overlap between toxic compounds in the different cell lines and found that 78% of toxic compounds in Hep G2 cells overlapped with compounds defined as toxic in U-2 OS cells (Figure 4B). Conversely, a higher response to the compounds was observed in the U-2 OS cell lines. According to the induction filter, more than 60% of the compounds displayed lower activity in the Hep G2 cell line (Table 1). Additionally, we observed that 87% of compounds with low activity in U-2 OS also had low activity in the Hep G2 cell line (Figure 4C). In summary, it appears that the Hep G2 cell line exhibits a smaller morphological response to the same compounds at the given concentration of 10μM, encompassing both their overall activity and toxicity.

Overall, the datasets show a high technical replication after filtering highly toxic and lower active compounds (Figure 3C; Table 1). When comparing the non-replicating compounds in both datasets we find as expected that they do not share a large overlap, as this will be determined by random technical variability (Figure 4D). We further made a direct quantitative comparison of the profiles across 564 non-toxic and highly active compounds in the different cell lines. To this end we used a variation of the percent replicating compounds, which we term percent pairing computed on the consensus profiles of each dataset.^31^ We found that more than 20% of the compounds compared have a correlating profile elevated from random samples in the dataset (Figures 4E and S9A; Table S5). Similar to the result from the feature space comparison across sites, the different datasets can be easily separated when visualizing them using UMAPs (Figure S9B).

### Analysis of morphological profiles

**Figure 5:**
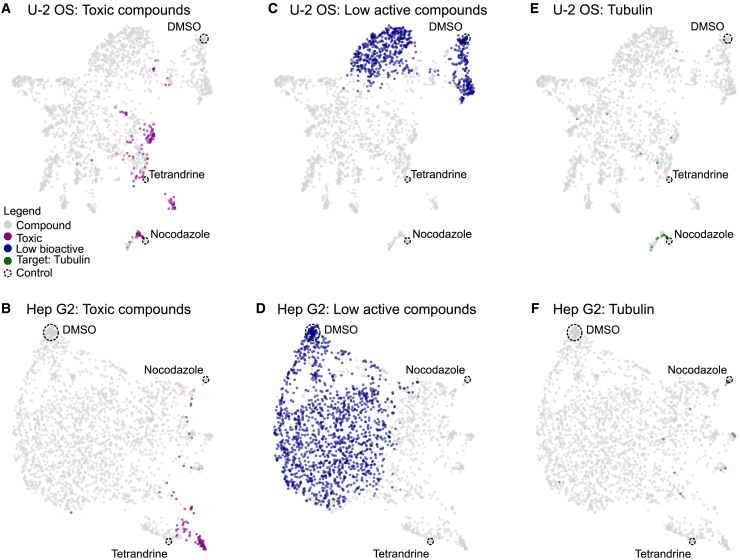
Visualization of morphological feature space based on FMP datasets Visualizations of morphological feature space using UMAP based on U-2 OS and Hep G2 cells after feature reduction and filtering of non-reproducible compounds. The location of the controls (DMSO, tetrandrine and nocodazole) are labeled using a dashed circle. (A and B) Magenta color label indicate compounds identified as toxic in U-2 OS (A) and Hep G2 cells (B). (C and D) Blue color label annotates compounds with low activity in in U-2 OS (C) and Hep G2 cells (D). (E and F) Green label for compounds annotated for tubulin as target in U-2 OS (E) and Hep G2 cells (F).

To visualize the wealth of data in the extracted profiles, we performed dimensionality reduction using UMAP. For a description of the entire datasets, we only filtered the datasets for the non-replicating compounds, including the toxic and low active compounds. We then proceeded to map the control compounds onto these visualizations (Figures S10A–S10F). We further projected the toxic and low active compounds as well one basic MOA based on compounds having tubulin as an annotated target onto these feature maps (Figures 5A–5F).

The visualization based on the U-2 OS cells reveals that toxic compounds are spread over a large part of the feature space. Toxic compounds are closely associated with both positive controls in particular with nocodazole in U-2 OS (Figure 5A). This observation confirms the toxic effect of nocodazole previously observed based on the cell numbers (Figure 3B; Table 2). For the Hep G2 cell line we can see that the overall smaller number of toxic compounds are located over a smaller area. In contrast to the result in the U-2 OS cells, the positive control nocodazole is not associated with any toxic compounds and forms a separate cluster (Figure 5B).

The negative control DMSO, is associated with low active compounds, with some compounds clustering closer to DMSO and many others spreading across a wider feature space in both cell lines (Figures 5C and 5D). This observation may suggest the presence of two distinct subsets of low-activity compounds: those with no activity and those exhibiting only low levels of activity. However, it is important to state not to over interpret UMAP visualizations as dimensionality reduction such as UMAPs are known to not necessarily preserve global structure in the data.^35^

Finally, in U-2 OS cells compounds with at least one target annotated against tubulin are closely associated with the positive control nocodazole (Figures 5E and S11A; Table S6) and compounds labeled as acting toxic at the tested concentration (Figures 5A and S11B). While the toxicity of compounds acting against tubulin is not unexpected,^36^ many other compounds in this specific cluster might not actually act against tubulin as severe cytotoxicity produces similar strongly correlating morphologies via diverse mechanisms that are not associated with specific MOAs (Table 2).^27^ Thus, in the case of U-2 OS cells at the tested concentrations it is therefore likely that the morphological profiles pick up on the very distinct morphology of dead or dying cells. It is important to note that the observed potential clusters constitute a hypothesis of MOA and require further experimental validation for a determination of their true MOA. In Hep G2 cells the compounds that are annotated with an MOA against tubulin fall also close to nocodazole (Figures 5F and S11C). In fact, all compounds of this cluster are directly implicated with tubulin or are published as tubulin inhibitors (Table S7). In contrast to the results in the U-2 OS cells no compounds in this cluster are annotated as acting toxic (Figures 5B and S11D). This indicates that at the tested concentration in Hep G2 cells the compounds in the cluster around nocodazole are truly associated with an MOA against tubulin rather than a general cytotoxic phenotype. This highlights the concentration dependent effect of compounds and their capacity for unspecific or off target effects that could be cell line specific.

Visualization of the feature space of the Hep G2 datasets from the other imaging sites, reveal that the relationship between the control compounds and different labeled compound classes is preserved, while the overall structure of the feature space is distinct between each imaging site (Figures S12A–S12I).

### Analysis of specific features using cellular senescence as example

**Figure 6:**
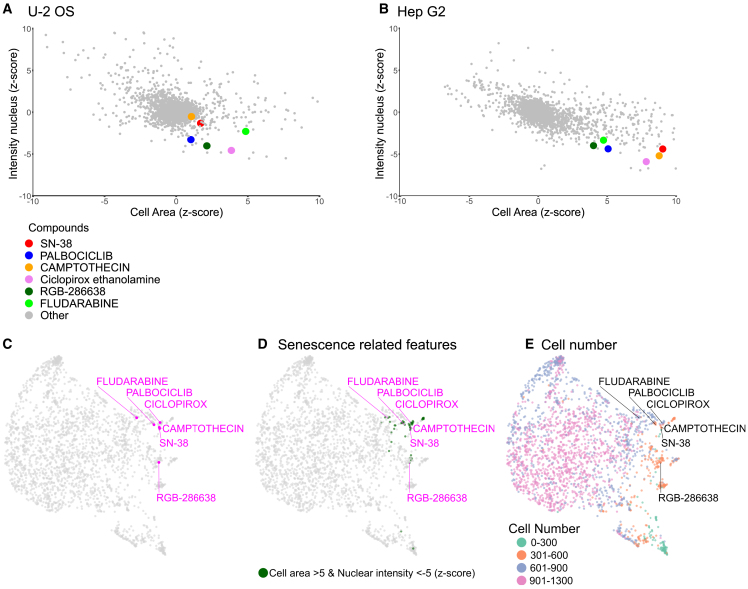
Assessment of cellular senescence using morphological profiling (A and B) Assessment of specific parameters indicative of cellular senescence based on U2 OS (A) and Hep G2 cells (B). (C–E) UMAP based on the FMP HepG2 dataset annotated for compounds associated with cellular senescence (C), senescence related features (cell area above *Z* score of 5 and nuclear intensity below *Z* score of 5 (D), as well as cell number bins (0–300, 301–600, 601–900 and 901–1300) (E).

Alternatively, to the analysis of the entire feature space using unsupervised machine learning we can also use specific features for measuring distinct cellular phenomena, such as cellular senescence. Cellular senescence is an arrest in the cell cycle with cells entering a stage without cell division.^37^^,^^38^ The morphological hallmarks are increased cell and nuclear size^39^ with abnormal nuclear morphology, in particular a decrease in density of the DAPI signal.^40^ We thus can use the intensity nucleus as well as the cell area measurements as a robust readout of cellular senescence. With this we can show that compounds known to induce cellular senescence^41^^,^^42^^,^^43^^,^^44^ indeed have increased cell size combined with a decrease in nuclear intensity in Hep G2 cells and to a lower extent in U-2 cells (Figures 6A and 6B).

This information can be also combined with analysis such as the visualization of the feature space using UMAPs. This reveals that some of the compounds associated with cellular senescence are closely associated with each other in the larger feature space (Figure 6C). Further annotating the compounds with senescence related features such as elevated cell area and reduced nuclear intensity (Figure 6D) as well as reduced cell number (Figure 6E) reveals further compounds that are clustering with these compounds as well as show similar selected feature responses (Figures 6D and 6E). Further analysis of eleven compounds that are closely associated with Ciclopirox (Table S8)^42^ show that two compounds namely triapine^45^ and mitoxantrone^46^ are directly implicated in cellular senescence. Further, three compounds such as SP2509,^47^ AHPN,^48^ and teniposide^49^ are indirectly implicated with cellular senescence via senescence related pathways.

## Discussion

We presented here a comprehensive Cell Painting dataset based on the EU-OPENSCREEN Bioactive compound set. The data were acquired from four different imaging sites and using two different cell lines. The advantage of this well characterized compound set lies in the diversity of selected compounds in terms of their chemical space, as well as well-annotated biological effects, including MOAs and targets. Such comprehensive annotation should not only facilitate downstream analysis and applications, such as MOA identification through unsupervised techniques (e.g., clustering) and other machine learning approaches,^50^^,^^51^^,^^52^^,^^53^ but more importantly, it also serves as a crucial reference for future morphological characterization of unknown compounds.

Our datasets have been generated with a full set of replicates across multiple imaging sites in three European countries. The data generation was preceded by an extensive selection, validation, and protocol optimization process toward establishing common processes across these different laboratories. Care was taken to standardize particularly on cell culture material. A systematic comparison revealed that these datasets exhibit high reproducibility and quality within the data produced at each imaging site, comparable to other published datasets.^17^^,^^54^ We envision that this dataset will not only be useful for assessing the influence of different technical confounding factors on the morphological profiles but can also be effectively used to develop methods for normalizing datasets acquired from different imaging sites. The knowledge gained will be important for developing strategies to better merge and analyze data from multiple sources, which is currently a major challenge in the field.^30^

The dataset is focusing primarily on the Hep G2 cell line, which is an established cellular model in high throughput screening.^55^ Hep G2 is a liver cancer cell line and, thus, aside from its relevance for liver diseases it is highly relevant as a cellular model for studying the toxic effect of small molecule compounds.^56^^,^^57^ To allow a comparison of our Cell Painting approach with other existing datasets, we further generated a dataset based on the U-2 OS cell line, as many datasets in the community have been generated using this cell line. We provide qualitative and quantitative comparisons between the morphological effects of the compounds on the different cell lines (Figures 4, 5, and 6).

We found that the Hep G2 cell line is overall less sensitive to toxic effects of compounds compared to U-2 OS at the relatively high used concentration of 10 μM for the tested compounds and 5 μM for the positive controls nocodazole and tetrandrine (Tables 1 and 2; Figure 4B). Important to note is that toxicity has been assessed based on the overall cell number per well and does not reflect a comprehensive assessment of toxicity. In addition, we have observed that the tested compounds at the given concentration show overall a smaller morphological response in Hep G2 (Figure 4C). Furthermore, the cell line due to its cellular morphology, particularly its compact clustered growth, can pose a challenge for image analysis and particularly feature extraction. This in part could also explain the observed lower overall activity of the tested Bioactive compounds at the used concentration.^28^

When visualizing the feature space using UMAPs, we were able to delineate in U-2 OS cells compounds annotated as tubulin modulators— closely associated with the positive control nocodazole, a known disruptor of microtubule assembly/disassembly^58^ (Figures 5E and S11A)— that were also associated with many toxic compounds (Figures 5A and S11B). Interestingly, in the same analysis, tubulin modulators in Hep G2, also closely associated with nocodazole (Figures 5F and S11C), showed no association with toxic compounds (Figures 5B and S11D). This indicates that in U-2 OS, the compounds at the given concentration show a profile associated with toxicity rather than a specific MOA. This highlights that small chemical compounds can produce varying effects due to MOA, polypharmacology, or off targets across different cell lines, and concentrations and emphasizes the need to perform Cell Painting in multiple cell lines and multiple concentrations for a more comprehensive and robust characterization. Finally, it underscores the potential of unbiased approaches such as Cell Painting to be used to exclude false positives. This is particularly important as the performance of supervised and semi-supervised machine learning approaches are highly dependent on accurate label information of their training data.^53^

The EU-OPENSCREEN Bioactive compound set is part of a much larger compound collection, currently containing more than 100,000 compounds. The largest library of this collection is the ECBL, an open-source compound library based on commercially available compounds designed to cover a wide and diverse chemical space. The compounds within the ECBL are continuously characterized for their bioactivity. Furthermore, EU-OPENSCREEN is collecting novel compounds synthesized from academic researchers from around the world in the European Academic Compound Library (EACL). Currently, this Cell Painting consortium is applying the presented Cell Painting approach to both the ECBL as well as the EACL. On the one hand, the presented dataset based on the Bioactive compounds served as an important milestone to the consortium to validate the approach and the feasibility to apply it to a much larger compound set. On the other hand, the very well characterized Bioactive dataset will serve as an important foundation and reference map for discovery of novel compound properties and MOAs within the ECBL and EACL.

Finally, the Cell Painting project is part of a much larger effort for a comprehensive characterization of the provided compounds based on the EU-OPENSCREEN Bioprofiling project. Here, general physico-chemical properties such as solubility, light absorbance, and fluorescence as well as biological properties such as cell viability, anti-bacterial, and anti-fungal are tested using common assay panels. The data produced by Cell Painting and the wider Bioprofiling project will be provided open source to the scientific community in public data repositories such as the Cell Painting Gallery^20^ and Bioimage Archive^59^ with the morphological profiles and other numeric data fully integrated in dedicated databases such as the European chemical biology database (ECBD).^60^ These data will provide a rich source for powerful computational approaches^61^ that promise to unlock the hidden potential of many small chemical compounds and thereby will accelerate early drug discovery.

### Limitations of the study

Morphological profiling using the Cell Painting assay has shown great promise in the prediction of the bioactivity of compounds. The assay has been shown to be robust and yields highly reproducible profiles. This study has further demonstrated that data acquired using the same compounds from different imaging sites produce highly similar profiles and, most importantly, very similar downstream predictions of bioactivity. However, we have also shown that the profiles are not directly comparable between imaging sites. Further work is required to optimize and standardize the data acquisition and further development is necessary for creating computational methods for aligning the outputs of different laboratories.

As shown in this study, the bioactivity of compounds can be dependent on the used cell line with the bioactivity exhibiting difference between the two tested cell lines U-2 OS and Hep G2. A further important aspect is the concentration of the compounds. In this study, the tested compounds were applied at a concentration of 10 μM and the positive controls nocodazole and tetrandrine at a concentration of 5 μM. This limits the extracted information from this dataset; in particular, in Hep G2 at the tested concentration, many compounds show lower activity. Further screens testing multiple concentrations will be beneficial to fully resolve the bioactivity of compounds.

The prediction of mechanism of action is still a technically challenging process. The development and application of new machine learning and deep learning–based prediction models promise to accelerate this process. Finally, the predictions of bioactivity for each compound represent a hypothesis. Further orthogonal analysis and/or follow up experimental studies are required to validate these results.

## Resource availability

### Lead contact

Further information and requests for data and code should be directed to the lead contact, Christopher Schmied (schmied@fmp-berlin.de).

### Materials availability

This study did not generate new unique reagents.

### Data and code availability

#### Data

• Original images have been deposited at the Cell Painting Gallery^20^ as cpg0036-EU-OS-bioactives and are publicly available as of date of the publication at Cell Painting Gallery: https://cellpainting-gallery.s3.amazonaws.com/index.html#cpg0036-EU-OS-bioactives/.
• Aggregated profiles have been deposited at Zenodo and are publicly available as of the date of publication at Zenodo: https://doi.org/10.5281/zenodo.14776021.
• Processed profiles have been deposited at Zenodo and are publicly available as of the date of publication at Zenodo: https://doi.org/10.5281/zenodo.14776021.
• Annotations for the EU-OPENSCREEN Bioactive compound set are available via the Probes & Drugs portal^62^: https://www.probes-drugs.org/compounds/standardized#compoundset=353@AND.

#### Code

• All original code has been deposited at Github and is publicly available at Github: https://github.com/schmiedc/EU-OS_bioactives as of the date of this publication.
• All original code for data analysis has been also deposited with the exact version used, inputs, and outputs are available at Zenodo and are publicly available as of the date of publication at Zenodo: https://doi.org/10.5281/zenodo.14776021.

#### Additional information

• Quality control plots have been deposited at Zenodo and are publicly available as of the date of publication at Zenodo: https://doi.org/10.5281/zenodo.14776021.
• All plots for figures have been deposited at Zenodo and are publicly available as of the date of publication at Zenodo: https://doi.org/10.5281/zenodo.14776021.
• Any additional information required to reanalyze the data reported in this paper is available from the lead contact upon request.

## Acknowledgments

We want to thank Sonja Sievers and Arnaud Ogier for their reviews in the first project evaluation phase, Beth Cimini for critical feedback for the project, Utesch Tillmann for HPC support and maintenance, Ankur Kumar and Erin Weisbart for support in uploading the data to the Cell Painting Gallery, the FMP IT Department, particularly Ingo Breng, for support, Michael Ebner for critical feedback to the image figures, Edgar Specker for input toward the compound collection, and Nathaniel Smith for lively discussion concerning the data analysis.

This project was supported by the Leibniz-Forschungsinstitut für Molekulare Pharmakologie via the Integrated Project titled: “Machine Learning Enhanced Cell Morphology Profiling in Molecular Pharmacology” awarded to J.P.V.K., H.S., and C.S.

This project was funded by the German Federal Ministry for Education and Research under grant number AZA 16KX1816.

The selection of Bioactive compounds was supported by the Ministry of Education, Youth and Sports of the Czech Republic (LM2023052).

This work was supported by the 10.13039/100016343EGI DataHub and EGI Check-in services from 10.13039/501100021676Cyfronet and GRNET, provided from the EGI-ACE project (10.13039/501100007601Horizon 2020) under Grant number 101017567.

## Author contributions

Conceptualization, C.W., M.N., M.H.C., P.D., K.E.S., B.S., M.H., M.L., J.P.K., H.S., and C.S.; methodology, C.W., C.J.B., D.S., A.S., M.J.V., J.B., P.D., M.C.R., K.E.S., M.L., J.P.K., H.S., and C.S.; software, M.N., C.J.B., J.B., and C.S.; validation, C.W., M.N., C.J.B., D.S., and C.S.; formal analysis, M.N., C.J.B., D.S., P.P., and C.S.; investigation, C.W., A.S., M.J.V., J.B., M.C.R., R.F.G., O.G., and M.I.L.; resources, C.W., M.H.C., P.D., R.F.G., B.S., O.G., M.H., and M.I.L.; data curation, M.N., C.J.B., and C.S.; writing – original draft, C.W., H.S., and C.S.; writing – review and editing, C.W., M.N., C.J.B., A.S., M.J.V., J.B., M.H.C., P.D., M.C.R., R.F.G., P.P., C.S., O.G., M.H., M.I.L., M.L., J.K., H.S., and C.S.; visualization, M.N., C.J.B., D.S., and C.S.; supervision, C.W., J.B., P.D., M.C.R., R.F.G., K.E.S., O.G., M.H., M.I.L., M.L., J.P.K., H.S., and C.S.; project administration, C.W., M.H.C., K.E.S., B.S., and C.S.; funding acquisition, C.S., M.H.C., B.S., J.P.K., H.S., and C.S.

## Declaration of interests

The authors declare no competing interests.

## Declaration of generative AI and AI-assisted technologies

During the preparation of this work the author(s) used scienceOS (https://www.scienceos.ai/) to identify references for targets and potential MOAs for selected compounds. After using this tool, the author(s) carefully reviewed all proposed references (i.e., checked that the reference exists, reference is valid for the text, read paper, and validated correct citation) and take(s) full responsibility for the content of the publication.

## STAR★Methods

### Key resources table

**Table:** 

REAGENT or RESOURCE	SOURCE	IDENTIFIER
**Chemicals, peptides, and recombinant proteins**		

Bioactives compound library	EU-OPENSCREEN	Probes & drugs: https://www.probes-drugs.org/compounds/standardized#compoundset=353@AND
Hoechst 33342	ThermoFisher Scientific	H3570
Concanavalin A Alexa Fluor 488	ThermoFisher Scientific	C11252
Wheat Germ Agglutinin Alexa Fluor 555	ThermoFisher Scientific	W32464
Phalloidin Alexa Fluor 568	ThermoFisher Scientific	A12380
MitoTracker Deep Red FM	ThermoFisher Scientific	M22426
SYTO 14	ThermoFisher Scientific	S7576

**Deposited data**		

Original images: cpg0036-EU-OS-bioactives	This study	Cell Painting Gallery: https://cellpainting-gallery.s3.amazonaws.com/index.html#cpg0036-EU-OS-bioactives/
Aggregated profiles	This study	Zenodo: https://doi.org/10.5281/zenodo.14776021
Processed profiles	This study	Zenodo: https://doi.org/10.5281/zenodo.14776021
Annotations for the EU-OPENSCREEN Bioactive Compound Set	Skuta et al.^62^	Probes & Drugs: https://www.probes-drugs.org/compounds/standardized#compoundset=353@AND
Quality control plots	This study	Zenodo: https://doi.org/10.5281/zenodo.14776021
Plots for figures	This study	Zenodo: https://doi.org/10.5281/zenodo.14776021
JUMP-CP compounds ID	Chandrasekaran et al.^16^	Github: https://github.com/jump-cellpainting/datasets/blob/main/metadata/compound.csv.gz
Annotations from the Broad Drug Repurposing hub	Corsello et al.^63^	https://repo-hub.broadinstitute.org/repurposing#download-data

**Experimental models: Cell lines**		

Human: Hep G2 cells	ATTC	HB-8065
Human: U-2 OS cells	DSMZ	ACC 785

**Software and algorithms**		

Analysis code	This study	Github: https://github.com/schmiedc/EU-OS_bioactives
Exact version of analysis code with inputs and outputs	This study	Zenodo: https://doi.org/10.5281/zenodo.14776021
CellProfiler version 4.1.3	Stirling et al.^8^	https://cellprofiler.org/previous-releases
JUMP analysis pipeline version 3	Chandrasekaran et al.^16^	Github: https://github.com/broadinstitute/imaging-platform-pipelines/tree/master/JUMP_production
HBOS filter in pyod version 1.0.9	Rezvani, Bigverdi, and Rohban,^25^ Zhao, Nasrullah and Li^64^	https://pyod.readthedocs.io/en/latest/index.html
python version 3.9.16	Rossum et al.^65^	N/A
pycytominer version 0.2.0	Serrano et al.^26^	https://github.com/cytomining/pycytominer
Precent replicating score	Way et al.^31^	N/A
Percent matching	Cimini et al.^17^	Github: https://github.com/carpenter-singh-lab/2023_Cimini_NatureProtocols/blob/main/README.md
Percent matching across modality	Cimini et al.^17^	Github: https://github.com/carpenter-singh-lab/2023_Cimini_NatureProtocols/blob/main/README.md
Induction filter	Christoforow et al.^23^	N/A
KNIME version 5.1.0	Berthold et al.^66^	www.knime.com
R version 4.3.1	R Core Team^67^	https://www.r-project.org/
R ggplot2 package version 3.4.3	Wickham^68^	https://ggplot2.tidyverse.org/
Uniform Manifold Approximation and Projections	McInnes et al.^29^	https://umap-learn.readthedocs.io/en/latest/
Fiji	Schindelin et al.^69^	https://fiji.sc/

**Other**		

PhenoPlate 384-well	Revvity	Cat# 6057308
Opera Phenix	Perkin Elmer (now Revvity)	https://www.revvity.com/de-en/category/high-content-screening-instruments
Operetta CLS	Perkin Elmer (now Revvity)	https://www.revvity.com/de-en/category/high-content-screening-instruments
Yokogawa CV8000	Yokogawa	https://www.yokogawa.com/de/solutions/products-and-services/life-science/high-content-analysis/cv8000/
BioTek EL406 or 405TS	BioTek (now Agilent)	https://www.agilent.com/en/product/microplate-instrumentation/automated-liquid-dispensing-handling/automated-microplate-washers-dispensers
Multidrop	ThermoFisher	https://www.thermofisher.com/de/en/home/life-science/lab-equipment/microplate-instruments/multidrop-dispensers.html
Blue Washer	BlueCatBio	https://www.bluecatbio.com/products/bluewasher/
Integra VIAFLO 384	Integra	https://www.integra-biosciences.com/global/en/electronic-pipettes/viaflo-96-viaflo-384
CERTUS FLEX liquid dispenser	Fritz Gyger AG	https://www.fgyger.ch/certus-flex/certus-flex-features-2/?lang=en
ECHO 550 or 650	Beckman-Coulter	https://www.beckman.de/liquid-handlers/echo-acoustic-liquid-handlers
Biomek i7	Beckman-Coulter	https://www.beckman.de/liquid-handlers/biomek-i7

### Experimental model and study participant details

#### Cell culture

Hep G2 cells (ATCC, HB-8065) were cultured in Roswell Park Memorial Institute Medium (RPMI 1640, Gibco, 61870044) supplemented with 10% (v/v) fetal bovine serum (Sigma Aldrich, S0615). U-2 OS cells (DSMZ, ACC785) were cultured in Dulbecco’s Minimum Essential Medium (DMEM, Gibco, 61965026) also supplemented with 10% FBS. The cell lines were tested for Mycoplasma using a luminescence-based MycoAlert kit (Lonza, LT07-418) and maintained at 37°C under 5% CO2. When cells reached a confluence of 70–80%, cells were washed with DPBS (Gibco, 14190250), dissociated with Trypsin-EDTA, 0.05% (Gibco; 25300025) and reseeded into a new cell culture flask with fresh complete medium or seeded for experiments.

#### Cell seeding

Hep G2 and U-2 OS cells are counted and seeded into 384 well plates (PhenoPlate 384-well, PerkinElmer, 6057328) using a Biotek or Multidrop microplate dispenser. Cells are seeded in a volume of 40 μL per well with 2000 cells/well and 700 cells/well, respectively and kept at room temperature for at least 30 min to aid homogeneous spreading. Plates were incubated for 24 h at 37°C at 5% CO2 atmosphere to allow for cell attachment and propagation. Compounds were transferred from library plates to cell plates using Echo 650 acoustic dispenser or Biomek I7 liquid handler. Plates were incubated for another 24 h at 37°C at 5% CO2 atmosphere.

### Method details

#### Bioactive Set

The EU-OPENSCREEN Bioactive Compound Set comprises 2,464 compounds selected utilizing data from the Probes & Drugs Portal (P&D)^70^ ver. 07.2018.^62^ P&D is a hub for the integration of high-quality bioactive compound sets with a focus on chemical probes and drugs. The set was created by the combination of manually selected high-quality chemical tools, such as chemical probes from the Structural Genomics Consortium [Structural Genomics Consortium, https://www.thesgc.org/] or Chemical probes portal,^71^ and an automatically generated set of, predominantly, target-selective compounds (prioritizing such with known mechanism of action), in order to achieve wide proteome coverage. The EU-OPENSCREEN Bioactive Set contains 385 compounds labeled as chemical probes and 681 as approved drugs. It is also a part of the P&D compound set list (probes-drugs.org/compoundsets) and therefore, can be accessed/worked with at P&D (Probes & Drugs: https://www.probes-drugs.org/compounds/standardized#compoundset=353@AND). The full list of compounds with basic annotations is a part of the supplementary material (Table S9).

#### Compound transfer

The EU-OPENSCREEN Bioactives Compound Set comprises seven 384 well plates with randomly distributed compounds located in column 1–22. Controls are located in column 23 and 24, which include DMSO (0.1%) as vehicle control as well as Nocodazole (5 μM) and Tetrandrine (5 μM) as reproducibility control. The concentration of Tetrandrine was adjusted based on cell viability data from the different imaging sites over different concentrations. 5μM was selected for Tetrandrine as the concentration for the control, as it provides the strongest profile with the lowest possible toxicity for all partner sites (Viability >0.4). Nocodazole is cytostatic (but not necessarily toxic) in the whole concentration range and was therefore adjusted to the concentration of Tetrandrine. Compound screen was performed at 10 μM in four replicates. For the replicates we used a new independent cell seeding event (biological replicate).

#### Cell Painting

The Cell Painting staining protocol is based on Bray et al. 2016^1^ with minor modifications. The protocol was adjusted by each site according to the available equipment (Table S10). In general, the medium was first aspirated from the plates to a residual volume of 10 μL. Subsequently 30 μL of MitoTracker (Invitrogen, M22426) solution in pre-warmed medium were added to the cells with a final concentration of 500 nM and incubated for 30 min at 37°C. For fixation, the MitoTracker solution was removed and replaced with 30 μL paraformaldehyde (4%, Roth, 0335) and incubated in the dark at room temperature (RT) for 20 min. After fixation, cells were washed with 70μL PBS and permeabilized by adding 30 μL of 0.1% (v/v) Triton X-100/PBS (Sigma Aldrich, T8787) solution to each well for 20 min at RT. Triton X-100 was removed followed by two washes with 70 μL PBS. Cells were then stained for 30 min at RT in the dark with of the staining solution containing HOECHST 33342 (Invitrogen, H3570), SYTO14 green (Invitrogen, S7576), Concanavalin A/Alexa Fluor 488 (Invitrogen, C11252), Wheat Germ Agglutinin (WGA)/Alexa Fluor 555 (Invitrogen, W32464) and Phalloidin/Alexa Fluor 568 (Invitrogen, A12380) in PBS with 1% (m/v) BSA (Sigma Aldrich, A7030). Adding 30 μL of the staining solution to each well resulted in the final well concentration of 4 μM HOECHST, 25 μg/mL Concanavalin A, 3 μM SYTO14, 1U/ml Phalloidin and 1.5 μg/ml WGA. Finally, cells were washed three times with 70 μL PBS and sealed with adhesive foil. Plates were stored in the dark at 4°C until image acquisition.

#### Assay optimization and standardization

We employed an extensive evaluation and validation process to select suitable imaging sites for performing the cell painting assay and achieve standardization and comparability.

#### First validation phase: Selection of imaging sites

An initial protocol based on an established Cell Painting Protocol was first optimized at the FMP site (Data S1: Cell Painting assay). We then invited imaging sites in an open call to submit a proposal and validation data. This initial proposal requires an estimation of costs and duration as well as information about the available instrumentation and a track record of performing automated screening assays.

To generate the validation data the developed protocol with the standardized staining and cell culture protocol was shared with imaging sites. To achieve further standardization and optimal comparability across the sites key reagents were shared. Reference compounds were prepared and aliquoted from the same lot and distributed to all sites. The same cell culture serum lot was acquired and provided to all sites. Finally, the cells were prepared and distributed by the FMP site.

The evaluation of the proposals and the validation data was performed in two stages based on pre-defined criteria by two external reviewers (Data S1: Evaluation Criteria, Data S1: Evaluation scoring). The reviewers were selected based on their expertise in the field of image-based screening and early drug discovery. In the first stage the provided data was evaluated based on how well the candidate sites implemented the protocol, the overall quality of the generated data, as well as for intra-as well as inter-plate consistency (Data S1: Evaluation stage 1). To pass the first evaluation more or equal to 65% of the first selection criteria needed to be reached. In the second stage, the candidate sites that passed the first criterion were evaluated for assay cost, the duration for screening 100,000 compounds, clarity of the proposal as well as a track record (Data S1: Evaluation stage 2). The score of the assay validation was considered with a factor of 10% in the second evaluation. Based on the participant’s final score four imaging sites were selected for performing the assay on the EU-OPENSCREEN Bioactive Compound Set.

#### Second evaluation phase: Protocol optimization

To reduce variability across sites we implemented additional measures and experiments. We reasoned that the main sources of variability observed could originate from differences in compound preparation, the cell culture, staining methods, or microscopy.^30^

To assess the effect of the microscopy on the result of our Cell Painting assays we performed an additional validation experiment. The FMP site shared a fully stained plate with the other three partner sites. We further developed a microscopy guide for the imaging sites so that the final images were in the same intensity range in each channel. In this experiment, we found no improvement in comparability across sites. To address any variability stemming from differences in compound preparation we distributed pre-prepared assay-ready plates (compound plates). This also did not improve the comparability. Thus, differences in cell culture as well as staining drive this variability. To exclude variability due to cell culture and staining material, we acquired, prepared and distributed the same serum lot, cells, and lots of fluorescent dyes centrally.

#### Image acquisition

Cell images were acquired using automated confocal microscopes equipped with water-immersion 20x objectives (1.0 NA). Offsets were determined for each cell line and kept constant throughout the experiment. For each well of the 384 well plate, nine fields in a 3 × 3 array, located in the center of the well, were imaged using 2x binning and four fluorescence channels to capture HOECHST 33342, Concanavalin A and SYTO14, Wheat Germ Agglutinin and Phalloidin, as well as MitoTracker. Excitation and emission wavelengths of these four channels vary based on the imaging system used by the different partner sites (Table S11).

#### Image feature extraction

We used CellProfiler version 4.1.3^8^ with the JUMP analysis pipeline version 3,^54^ original available here at Github: https://github.com/broadinstitute/imaging-platform-pipelines/tree/master/JUMP_production) for feature extraction. A total of 2977 features were extracted from each segmented cell in all fields per well. The pipeline was executed in parallel on 5 nodes of our high-performance computing cluster (HPC) (Table S12).

We adjusted the feature extraction pipeline for our four-channel dataset (DNA, ER, AGP and Mito). The illumination function was calculated on the individual channels with a median filter set to a kernel size of 20 px. The function was computed on all images across cycles with rescaling. The illumination function was applied using a division. No images were removed based on the image quality control of the CellProfiler workflow.

Nuclei in the DNA channel were segmented with a global minimum cross-entropy threshold using a threshold smoothing scale of 1 and a threshold correction factor of 1. The lower and upper bounds of the threshold were set to 0.005 and 1.0 respectively. No log transform of the image intensity values was performed before thresholding. The shape of the objects was used to separate objects with a smoothing filter using a radius of 10 px applied before separation. Local maxima with a distance smaller than 8 px were suppressed. Holes were filled after de-clumping. Nuclei with a diameter of 15 px - 90 px were kept in U-2 OS and Hep G2 cells.

Cells were segmented based on the ER channel using a marker-controlled watershed using the segmented nuclei as input. A global intensity-based threshold using the Otsu thresholding method was used to compute a three-class threshold, assigning the pixels of the middle class to foreground. No smoothing was applied to the images. The threshold correction factor was set to 0.7. The lower and upper bounds of the threshold were fixed to 0.005 and 0.6 respectively. A log transformation was applied before thresholding. Cell and nuclei objects that were touching the image border were filtered for some of the feature computation. To create a mask of only the cytoplasm the nuclei were subtracted from the cell mask.

The following image features were computed based on the AGP, DNA, ER, and Mito channel within the nuclei, cell, and cytoplasm mask. All correlation metrics with a threshold of 15% of the maximum intensity. The Faster method was used to compute a Costes thresholding. Granularity was measured with a subsampling factor of 0.5 and a subsampling factor of 0.5 for background reduction. The radius of the structuring element was set to 10 and the range of the granular spectrum set to 16. The intensity in the illumination corrected images was measured. The intensity distribution within the objects was computed by scaling the bins and using 4 bins.

Textures were measured in nuclei, cells and cytoplasm in the DNA, ER, Mito channel using 256 Gy level bins with a scale of 3, 5, and 10. The size and shape of nuclei, cells, and cytoplasm including Zernike features were extracted. The number of cell neighbors was measured within a 5 px distance including cells that were touching the image border. Further the number of all adjacent cells were measured including cells that were touching the image border. The number of nuclei neighbors were measured within a 1 px distance including nuclei that were touching the image border. For the Mito channel the Neurites feature type score was computed enhancing the tubeness enhanced Mito channel with a smoothing scale of 1. Based on the tubeness enhanced Mito image, the intensity distribution of cells with nuclei as center, the cytoplasm with nuclei as center was computed by scaling the bins and using 16 and 20 bins with a maximum radius of 200 px.

On the tubeness enhanced Mito channel a global minimum cross-entropy threshold was applied using a smoothing scale of 1.3488 and a correction factor of 1.0. The lower and upper bounds for the threshold were set to 0.0 and 1.0 respectively. No log transform was performed before the thresholding. On the mask of the Mito channel a skeletonization algorithm was applied with filling in small holes of maximum 10 px. Finally, skeleton features were extracted.

Overall image intensities were extracted from the illumination corrected images. Background in each channel was computed in the image content outside of the segmented objects.

#### Single cell filter

Before aggregation, the measurements for individual cells were filtered to remove cells with any missing or infinite values. Furthermore, an HBOS filter was performed to remove objects that had a feature vector that was different from the distribution of features over the entire plate.^25^ In our tests, this was well suited to particularly remove artifacts in segmentation as well as dead cells. The outlier selection was computed with the HBOS function of pyod^64^ version 1.0.9 with python^65^ version 3.9.16. The histogram was computed using a static number of bins of 10, an alpha of 0.1, the flexibility parameter (tol) set to 0.5, and the proportion of outliers (contamination) set to 0.1. The HBOS model was then applied to classify each individual feature vector into outlier and non-outlier.

After the outlier, missing and infinite values filters, median values were computed for each well based on up to nine fields per well. Note that for some fields no segmentation was achieved, or they were removed in the filter step before data aggregation. Some particular toxic compounds had no extracted features over all replicates; these were counted toward the toxic compounds number.

#### Similarity search

The structural overlap between the EU-OS bioactives compound collection (www.ecbd.eu, library “Bioactives”) and the compound investigated in the JumpCP project (Github: https://github.com/jump-cellpainting/datasets/blob/main/metadata/compound.csv.gz) was determined as follows: the compound structures were read into KNIME software and the ECFP-4 fingerprints generated using the CDK community node extension. Using the “Similarity Search” node, for each entry of the bioactives dataset the identifier of the most similar compound in the JumpCP dataset was listed if its tanimoto similarity was equal or greater than 95%.

#### Normalization, feature reduction and profile aggregation

We used pycytominer^26^ version 0.2.0 in python 3.9.16 on Ubuntu 22.04.3 LTS for further processing of the profiles. The image features per well were normalized per plate to the DMSO controls using the robust median absolute deviation function (mad_robustize). The epsilon value was set to the default value of 1e-06. Feature reduction was performed by first removing columns with NaN values. Then features with a low variance were removed via a variance frequency cut-off of 0.1 and a variance unique cut-off of 0.1. Feature outliers were removed with the outlier cut-off set to 100. Finally, features with high correlation were reduced using a correlation threshold of 0.9. For aggregating the profiles over the four replicates the median function was used.

#### Toxicity filter

We determined toxic compounds as these have been shown to produce highly similar features with unspecific MOAs.^27^ We first compute the consensus median cell number for each well per plate over the four replicates. We then defined compounds as toxic with per well consensus cell count smaller than the median of the population of the entire dataset subtracted by 2.5 standard deviations of the population.

#### Activity filter

The percent of replicating compounds over the compound set after the toxicity filter was initially ranging from 60.1 to 77.5% (Figures S13A–S13E). We also observed that many of the compounds in both cell lines gave very small responses in their morphological profiles compared to the DMSO negative control. We suspected that many of the compounds with small phenotypic response in the specific cell line also exhibit low reproducibility. To determine compounds with lower activity we applied an induction filter.^23^ To determined induction first median features per compound over the four replicates were computed. Features were defined responding when deviating three times from the median absolute deviation from the median of the DMSO controls. For each compound the fraction of active features was then computed and a threshold of 5% applied to define a compound with an active response.

#### Plate quality controls

The aggregated data files containing the median values for each well per plate were further aggregated into a single dataset using a KNIME workflow. For visualization of plate artifacts five CellProfiler features were selected: Metadata_Object_Count, reflecting the number of detected cell objects. Further the mean fluorescence intensity values of following compartments and stains, reflecting the dispense quality of the dyes: Nuc_Intensity_MeanIntensity_DNA (HOECHST 33342), Nuc_Intensity_MeanIntensity_ER and Cyto_Intensity_MeanIntensity_ER (Concanavalin A & SYTO14), Cyto_Intensity_MeanIntensity_AGP (Phalloidin & Wheat germ agglutinin), Cyto_Intensity_MeanIntensity_Mito (Mitotracker Deep red).

The data for each plate and feature was visualized using heat maps that were generated within a KNIME^66^ workflow using R^67^ snippets and the R library “ggplot2”.^68^ KNIME version 5.1.0, R version 4.3.1, R ggplot2 package version 3.4.3 was used. The generated plots were then transferred into the integrated reporting tool of KNIME, and assembled into a single printable report file for each dataset. Heatmaps of Metadata_Object_Count were scaled as follows: the global maximum and median were determined, and rounded up to two digits. The color yellow was assigned to the median object count, blue to the maximum, red to zero objects. This way it is possible to spot differences in absolute cell number that was dispensed across the different plates. Heatmaps of the mean fluorescence features were scaled as follows: for each plate, the values were divided by their median for data normalization, since we do not have plate controls at hand that are specific for those features. The color yellow was assigned to the median value, blue to 1.5-fold the median value, and red to 0.5-fold the median value.

The plots of the four technical replicates were combined on a single report page for each screened library plate. The median *Z* score values of these four technical replicates were added as a fifth heatmap. This way, it is possible to spot whether taking the median of the replicates would reduce plate artifacts that are visible on individual technical replicates.

#### Cell death analysis

For the analysis of dead cells in U2OS and Hep G2 two features “cell area” and “ratio cell width to length” were used. For classification of dead cells, the following thresholds were defined: cell area <1,000 μm^2^ and ratio cell width/cell length >0.83.

#### Dimensionality reduction

We used Uniform Manifold Approximation and Projections (UMAP)^29^ to create visualizations of the morphological feature space in 2D. For the batch control quality figures, we used the data filtered for toxic, lower activity and non-replicating compounds. To perform the UMAPs for the batch quality control we used the default settings using the Euclidean metric, with the number of neighbors set to 15 and the minimum distance set to 0.1. The data points in the visualizations were then labeled by their replicate or plate number.

For the analysis of the morphological features space, we used the data only filtered for non-replicating compounds leaving in the toxic as well as the lower active compound. The number of neighbors was set to 30 with a minimum distance of 0.1. We then labeled on the UMAP visualizations the control compounds (DMSO, Nocodazole and Tetrandrine). The data points were then labeled for toxic as well as lower active compounds. For the basic MOA analysis for compounds, acting against Tubulin we did a basic search and asked if a single target in the annotated targets of any compound contained the string ‘Tubulin’.

#### Senescence analysis

For the analysis of cellular senescence, we extracted the cell area as well as the nuclear intensities for all compounds of each FMP dataset. For this analysis we computed Z-Scores based on the median and median absolute deviation of the entire plate. The normalized parameters were then plotted against each other in a 2D dot plot. Finally, we labeled compounds that are known to induce cellular senescence^41^ (e.g., PALBOCICLIB, CAMPTOTHECIN and SN-38 an active metabolite of a CAMPTOTHECIN analog) on these plots.

#### Image figures

Image figures were prepared with established processing and image visualization standards^72^^,^^73^ using Fiji.^69^ In detail for the image figures, randomly selected wells from the negative and positive controls were used. For Figures 2C and 2D the fifth (middle) field of the selected well was used. For Figures S1 and S2 the first field of the selected well was used. The individual images for each channel were corrected for illumination using the same method as the HPC CellProfiler cluster workflow. The lower bound of the brightness contrast function was then set in all treatments to an empirically determined camera background in the DMSO treated images within each individual channel. Three large rectangular ROIs were drawn in an area of the image without cells. The mean gray value was measured in the ROIs and an average was computed. The upper bound was adjusted based on the brightest treatment in each individual channel and applied over all treatments to ensure intensity values can be compared over the treatments within a single cell line (Tables S13 and S14).

After brightness and contrast adjustments, the images were converted to 8-bit and saved as PNG. For an overview of the DNA, AGP and Mito channels were merged with Cyan, Magenta and Green LUTs respectively. A ROI for the inset was chosen in the middle of the field of view. ROIs as well as scale bars are shown on the overview images.

### Quantification and statistical analysis

Quantitative metrics were computed using python version 3.9.16. Details of the metrics can be found in the figure legends in particular for the raw metric graphs in the supplements.

#### Percent replicating

The percent of replicating compounds was computed based on the precent replicating score developed in the JUMP-CP consortium.^31^ For each compound the pairwise Pearson correlation over all available replicates was determined and a median replicate correlation computed. To compute a null distribution, 10,000 random samples of four randomly chosen compound feature vectors (non-replicates) were drawn from the dataset. The pairwise Pearson correlation of these feature vectors were computed and used to calculate a median non-replicate correlation. Compounds were defined as replicating if their median replicate correlation was more than 95% of the null distribution computed from the samples of the median non-replicate correlation.

#### Percent matching and percent matching over datasets

Percent matching was implemented from Cimini et al. 2022.^17^ Annotation was generated by combining annotations from the Broad Drug Repurposing hub (https://repo-hub.broadinstitute.org/repurposing#download-data)^63^ with our annotations. Each MOA annotation on each compound was treated as a separate MOA. The MOAs were filtered for more or equal of 3 occurances. Precent matching over datasets was implemented from percent matching across modality.^17^

#### Percent paring

For comparing the profiles between the two cell lines, the percent replicating metric was computed over the corresponding compounds after toxicity, activity and reproducibility filtering. We used the 427 overlapping features of the cell lines after feature reduction and computation of consensus profiles (Figure 4A). To differentiate this metric from the percent replicating metric we changed the name of this analysis to percent pairing. The null distribution was computed over randomly selected compound pairs. The same compound pairs over the two different cell lines were defined as pairing if above the 95% correlation threshold defined on the null distribution.

## Supplemental information

Document S1. Figures S1–S13, Tables S10–S14, and Data S1

Table S1. Target classes of EU-OPENSCREEN Bioactive compounds

Table S2. Pathway classes of EU-OPENSCREEN Bioactive compounds

Table S3. Overlap of EU-OPENSCREEN Bioactive compounds overlap with JUMP-CP compounds

Table S4. Result of feature reduction of each dataset. Number of features before and after feature reduction

Table S5. Pairing compounds across Hep G2 and U-2 OS. Compound IDs and Probes & Drugs information of compounds that pair across the two tested cell lines

Table S6. Compounds in U-2 OS Nocodazole cluster. IDs and references for compounds in Nocodazole cluster based on the U-2 OS cell line

Table S7. Compounds in Hep G2 Nocodazole cluster. IDs and references for compounds in Nocodazole cluster based on the Hep G2 cell line

Table S8. Compounds in Hep G2 Ciclopirox cluster. IDs and references for compounds in Nocodazole cluster

Table S9. Probes & Drugs files of EU-OPENSCREEN Bioactive compounds. Compound IDs and their annotation in Probes and Drugs as well as the annotated targets of these compounds
